# Posterior occipital intramuscular hemangioma mimicking arteriovenous malformation: Case report

**DOI:** 10.1097/MD.0000000000014678

**Published:** 2019-03-15

**Authors:** Hao Chen, Baofeng Xu, Guangming Wang, Yunbao Guo, Kun Hou, Jinlu Yu

**Affiliations:** Department of Neurosurgery, The First Hospital of Jilin University, Changchun, China.

**Keywords:** arteriovenous malformation, intramuscular hemangioma, posterior occipital

## Abstract

**Rationale:**

Intermuscular hemangioma (IH) usually occurs in the muscles of the limbs and trunk, but can rarely occur in the occipital region. IH in the occipital region is easily misdiagnosed as arteriovenous malformation (AVM).

**Patient concerns:**

A 31-year-old woman had a right occipital mass for 5 months without pulsation.

**Diagnosis:**

Head computered tomography angiography (CTA) and digital substraction angiography (DSA) examinations showed that the lesion was mainly vascular, approximately 3 × 5 cm in size, and supplied by occipital arteries and the muscular branches of vertebral arteries. The venous drainage of the lesions communicated with the suboccipital vein plexus and the paravertebral vein in the venous phase, indicating AVM. Postoperative histological investigation showed the lesion was a hemangioma.

**Interventions:**

It was recommended for surgical removal. The surgery was carried out under general anesthesia. The lesion showed a clear boundary. The occipital artery touched the anterior margin of the lesion, was exposed and ligated, and was removed around the lesion. The lesion consisted of massive blood vessels, and the surrounding muscles were swollen, indicating IH.

After the lesion was removed, the normal muscle tissue around the lesion was also removed.

**Outcomes:**

The patient achieved a good recovery after surgery, and pathology confirmed IH. A postoperative 1-year CTA review was performed and showed partial residual, then the radiotherapy was recommended. She refused further radiotherapy, follow-up 2 years later showed no enlargement of the lesion.

**Lessons:**

Although IH rarely occurs in the occipital region, this can occur. Due to the complexity of the drainage veins in the occipital region, these IH are prone to misdiagnosis as AVM.

## 1 Introduction

Intermuscular hemangioma (IH) is a type of benign endothelioma that often occurs in the muscles of the limbs and in the body, but can rarely occur in the head and neck. IH occurring in the head and neck accounts for approximately 15% of all IHs. While they mainly occur in the masseter, trapezius muscle and temporalis, they can rarely occur in the posterior occipital area.^[1,2]^ The posterior occipital area is rich in blood-supplying arteries, including the occipital artery and the muscular branch of the vertebral artery, and its drainage veins are the paravertebral venous plexus and the inferior occipital venous plexus.^[3,4]^ A rare case of posterior occipital IH is reported in this paper. The case was characterized as an arteriovenous malformation (AVM) on imaging due to its abundant blood transport in the posterior occipital area. The performance and treatment of this case are reported, and the related literature is reviewed.

## 2 Case report

The patient, a 31-year-old woman, was admitted to the department of neurosurgery at The First Hospital of Jilin University due to a “right occipital mass for 5 months”. The patient was in good health, and her family members did not have similar lesions. A physical examination showed that the mass was in the right occipital region at the upper margin of the sternocleidomastoid muscle and exhibited slight tenderness without obvious pulsation or vascular murmurs during auscultation.

**Figure 1:**
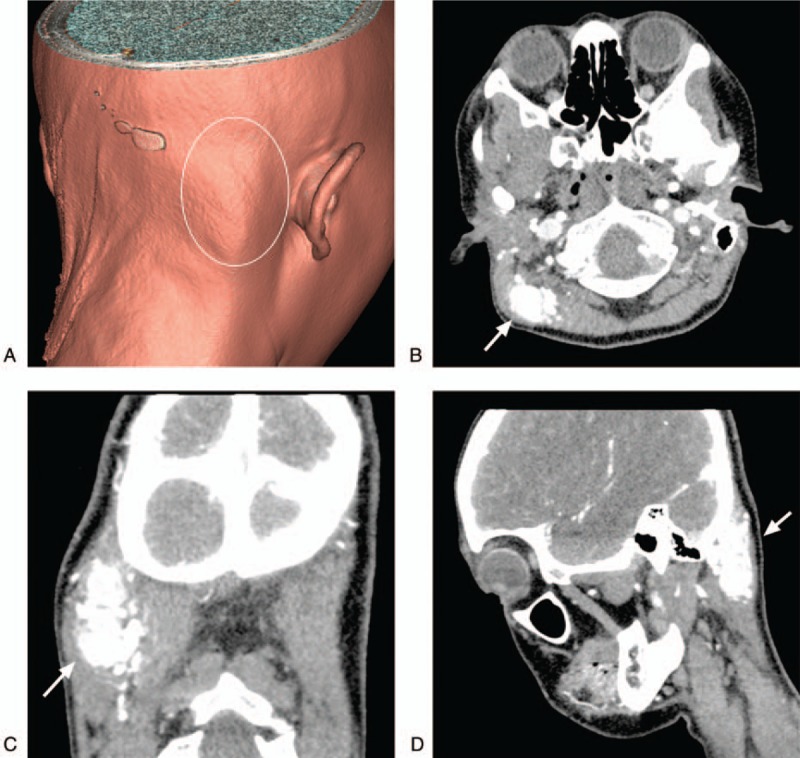
Preoperative CT and 3D reconstruction images. A: A 3D reconstructed CT showing the lesion in the right occipital region with a slight bulge in the skin (white circle). B–D: CT enhanced images of axial, coronal and sagittal planes showing that the lesions were mainly vascular in nature, presented as a “wool mass”, were located in a subcutaneous region, involved the muscles, and showed obvious enhancement (arrow).

**Figure 2:**
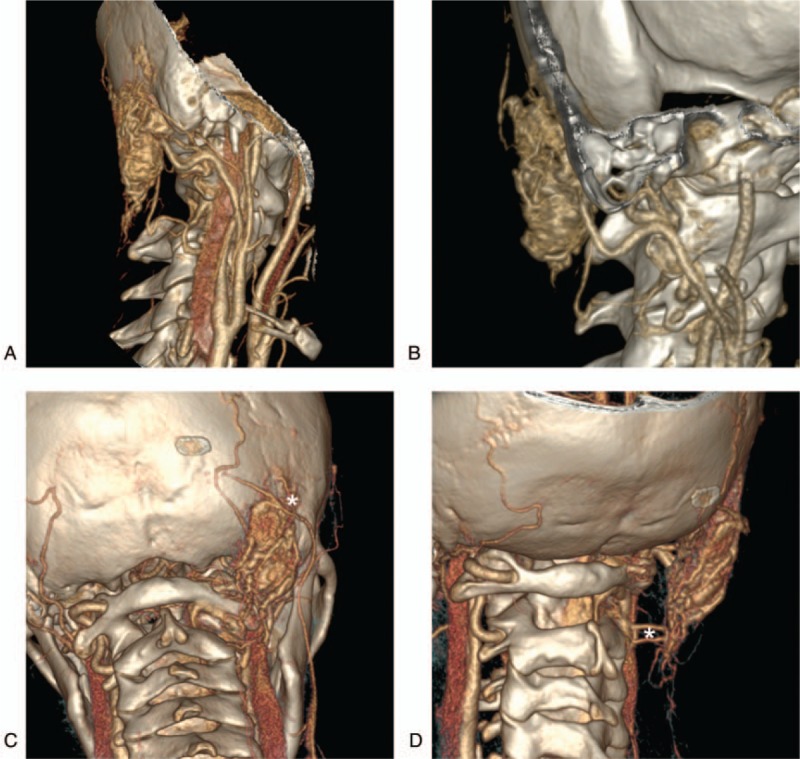
Preoperative CTA images. A–B: CTA after the reconstruction revealed the main blood supply arteries from multiple angles. The lesion size was approximately 3 × 5 cm, and it was mainly supplied by the occipital artery and the muscle branch of the vertebral arteries. C: The venous drainage of the lesion communicated with the suboccipital venous plexus at the anterior and posterior CTA, and it also communicated with the subcutaneous superficial vein. D: The venous drainage of the lesion communicated with the paravertebral venous plexuson anterior and posterior oblique CTA (white asterisk). CTA = computered tomography angiography.

A head CTA examination at admission suggested that the lesion was approximately 3 × 5 cm in size, was mainly composed of vascular components, presented as a “wool mass”, was located in a subcutaneous region, involved the muscles, and showed obvious enhancement. The lesion was mainly supplied by the occipital and muscular branches of the vertebral arteries. The venous drainage of lesion communicated with the suboccipital venous plexus and the paravertebral venous plexus, and the venous drainage communicated with the subcutaneous cervical superficial veins (Figs. 1 and 2).

**Figure 3:**
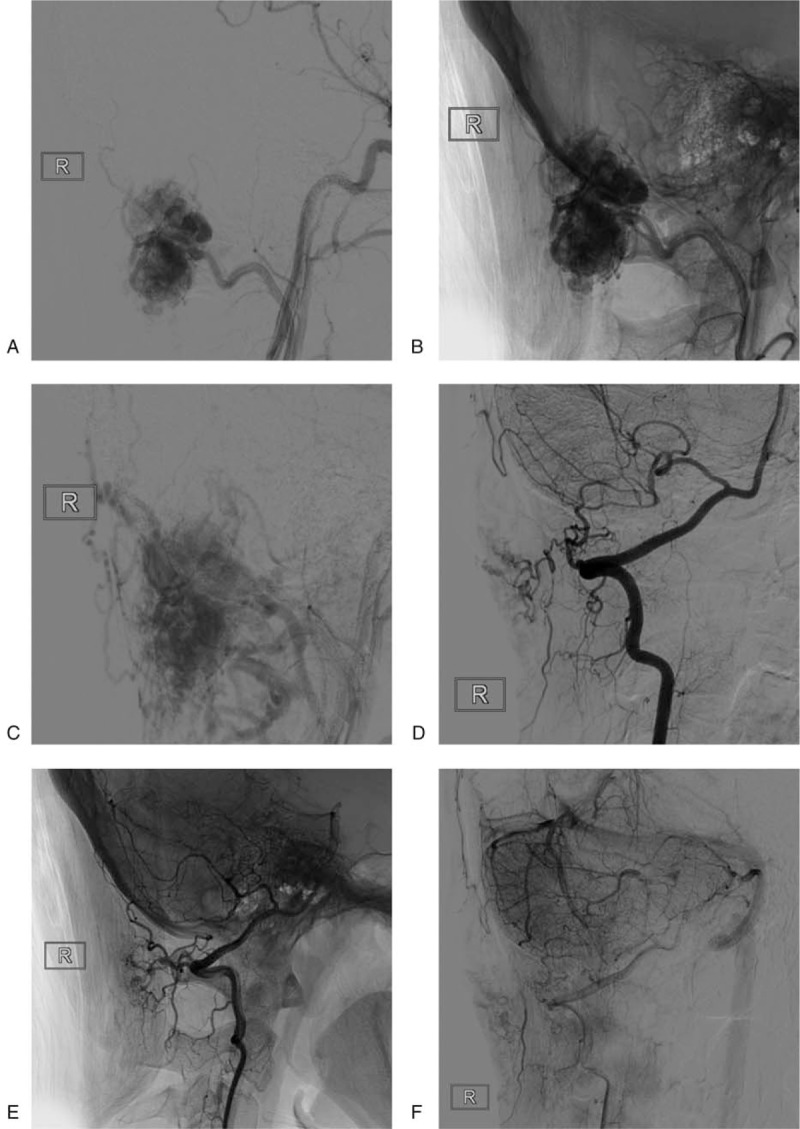
Preoperative DSA image. A–B: Right external carotid artery angiography showing a rich blood supply and obvious contrast staining, mainly from the occipital arteries. C: During the venous phase, the lesion can be seen draining into the suboccipital venous plexus and the paravertebral venous plexus. D–E: Vertebral artery angiography showing that the muscular branch of the vertebral artery supplied the blood, and the blood supply was low; F: Vertebral arteriography showing poor filling of the lesion during the venous stage. DSA = digital substraction angiography.

A further DSA examination revealed that the blood supply of the lesion was mainly from the occipital artery and less from the muscle branches of the vertebral arteries (Fig. 3). The lesion was considered an AVM based on its imaging characteristics and was recommended for surgical removal. The surgery was carried out under general anesthesia. The lesion could be touched when the occipital skin was incised during the operation. The lesion showed a clear boundary. The occipital artery touched the anterior margin of the lesion, was exposed and ligated, and was removed around the lesion. The lesion consisted of massive blood vessels, and the surrounding muscles were swollen, indicating IH.

**Figure 4:**
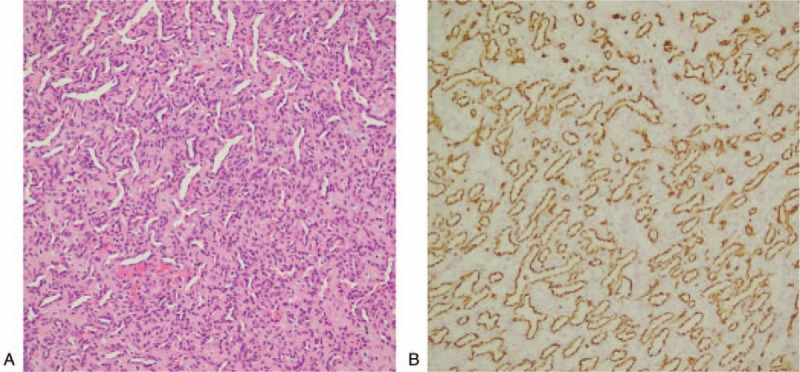
Pathological image of the lesion. A: HE staining showing the lesion almost purely consisted of capillaries with only rare cavernous components among the muscle. B: CD31 staining was positive in the vessel endothelium, indicating a clear diagnosis of intramuscular hemangioma.

**Figure 5:**
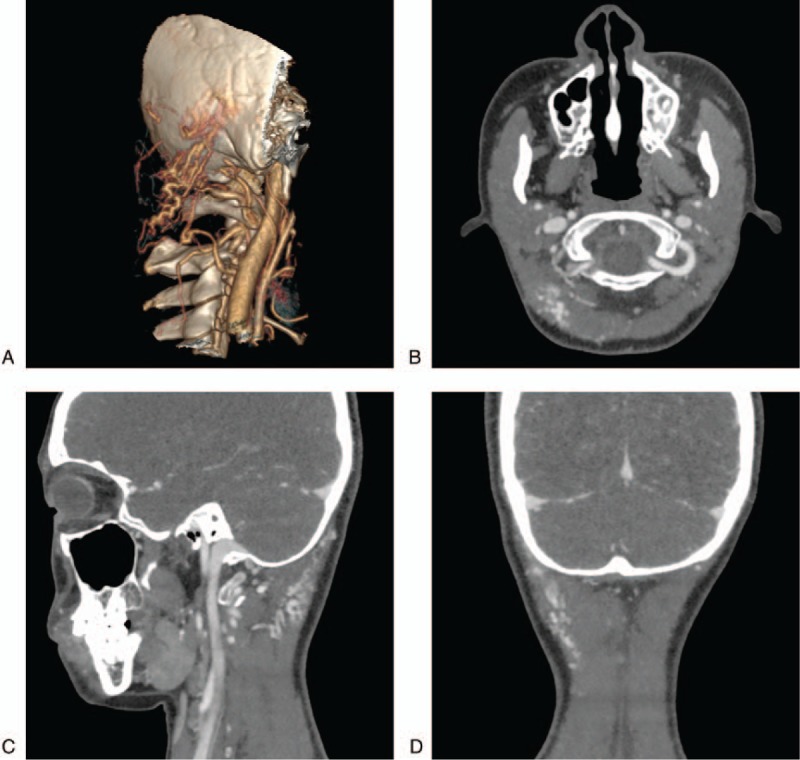
Postoperative review of CTA. A: Reconstructed CTA showing a small amount of residual lesions. B–D: Enhanced CT showing residual intramuscular hemangioma enhancement. CTA = computered tomography angiography.

After the lesion was removed, the normal muscle tissue around the lesion was also removed. The specimen was sent to pathology, and postoperative pathology confirmed that the lesion was IH accompanied by thrombolytic recanalization. HE staining showed that the lesion almost purely consisted of capillaries with only rare cavernous components among the muscles. CD31 staining was positive in the vessel endothelium, indicating that the lesion was an IH (Fig. 4). The patient achieved a good recovery after surgery and was discharged from the hospital. A head CTA review performed one year later showed partial residual IH (Fig. 5). The patient had no obvious discomfort, and the hemangioma could not be touched from the surface, and the patient was recommended for radiation therapy. The patient refused radiation therapy. Follow-up 2 years later showed no enlargement of the IH.

## 3 Ethics statement

Being a Case Report, our institution does not require formal Ethical Approval. Written informed consent was obtained from the patient and her husband for publication of this case report.

## 4 Discussion

IH is a type of benign endothelioma. The first reported case of IH is attributed to Liston in 1843, who called this entity an “erectile tumor”. IH is rare and often occurs in the muscles of the limbs and the trunk but only rarely occurs in the head and neck.^[1,2]^ Based on its pathological classification, IH is divided into a capillary type, a cavernous hemangioma type and a mixed type, the capillary type the most common.^[5]^ The IH, in this case, occurred in the right occipital muscle and was capillary type. Because this location is rare, there have been few such cases reported in the literature. Our case enriches our view of IH and indicates one more type of disease that should be considered in diagnoses of opisthotic-occipital lesions.

IH is rarely found in the lateral occipital region, which is a very critical region, mainly due to its complex arterial and venous structures.^[3,6]^ In the posterior occipital region, the arteries mainly include the occipital artery and the muscular branches of the vertebral artery, and the drainage veins are the paravertebral venous plexus and the suboccipital venous plexus. Its extensive venous drainage introduces great difficulty when diagnosing vascular diseases in this region.^[7]^ The IH, in this case, was mainly supplied by the occipital branches and the muscle branches of the vertebral artery and drained to the suboccipital venous plexus, paravertebral venous plexus and subcutaneous superficial vein. These characteristics of its blood flow share similarities with those of AVM that occur in this area.

However, postoperative pathology confirmed the lesion was an IH. A retrospective analysis of DSA images of the IH revealed differences between IH and AVM. Due to the tumor nature of the IH, different blood supplying arteries will supply different parts of the IH, and the IH can therefore only be partially clearly displayed on angiography of different blood supplying arteries. As shown in Figure 3, the IH showed different areas on occipital artery and vertebral artery angiography. However, in DSA images of an AVM, because different blood supply arteries are connected with the nidus of the AVM, the nidus of the AVM can be seen on arteriography of different blood supply arteries.^[8]^

IH has a rapid growth rate and is prone to recurrence and therefore requires active treatment.^[9]^ The treatments for IH include surgical resection, sclerotherapy, cryotherapy, and radiotherapy. Surgical resection is the best choice for treatment of this disease. Other methods can also be considered to alleviate pain, reduce depressive symptoms and reduce postoperative recurrence.^[9]^ IH have a rich blood supply. During surgery, the blood supplying arteries of the IH should first be controlled. In this case, the occipital artery was located at the anterior edge of the IH. It was ligated and removed around the IH. The IH was found to consist of massive blood vessels during the operation. The adjacent muscles were swollen, and postoperative pathology confirmed IH (capillaries).

A postoperative 1-year CTA review of this case showed partial IH residues. Reports in the literature indicate that the recurrence rate of IH after surgical resection is 9% to 28%, with recurrence rate 28% in mixed type, 20% in capillary type, and 9% in cavernous hemangioma type.^[10]^ Although radiotherapy and other methods are feasible for treating tumor recurrence after IH resection, as full a resection as possible should be achieved in the first operation. In this case, the IH was treated with radiotherapy because little residue remained.

Therefore, although IH rarely occurs in the lateral occipital region, this can occur. Due to the complexity of the drainage veins in the occipital area, IH is prone to be misdiagnosed as a vascular malformation on imaging. A lateral occipital IH should be surgically removed, and if there is any residual, radiotherapy should be recommended for.

## Author contributions

**Conceptualization:** Hao Chen and Yunbao Guo.

**Methodology:** Guangming Wang.

**Resources:** Baofeng Xu.

**Writing – original draft:** Yunbao Guo.

**Writing – review & editing:** Kun Hou and Jinlu Yu.
